# TRPV1 channel antagonist capsazepine alleviates morphine tolerance and morphine-induced neurotoxicity by preventing mitochondrial damage and apoptosis: an in vivo and in vitro study

**DOI:** 10.1007/s00210-025-04384-5

**Published:** 2025-06-23

**Authors:** Aysegul Ozturk, Ercan Ozdemir, Mustafa Ozkaraca, Ahmet Sevki Taskiran, Ahmet Altun

**Affiliations:** 1 Departments of Therapy and Rehabilitation, Vocational School of Health Services, Sivas Cumhuriyet University, Sivas, Turkey; 2 Departments of Physiology, Medicine Faculty, Sivas Cumhuriyet University, 58140 Sivas, Turkey; 3 Department of Pathology, School of Veterinary Medicine, Sivas Cumhuriyet University, Sivas, Turkey; 4 Departments of Pharmacology, Medicine Faculty, Sivas Cumhuriyet University, Sivas, Turkey

**Keywords:** Capsazepine, Morphine tolerance, Neurotoxicity, Mitochondrial damage, Apoptosis

## Abstract

Morphine is one of the drugs frequently used for severe pain in chronic diseases such as cancer, but long-term use leads to morphine tolerance. The mechanism of morphine tolerance is not yet fully understood. This study aimed to investigate the effects of the TRPV1 channel antagonist capsazepine (CPZ) on morphine tolerance and morphine-induced neurotoxicity, mitochondrial damage, and apoptosis by in vivo and in vitro methods. Thirty-six male Wistar Albino rats, aged 12–14 weeks (weight 230–250 g), were included in the study. To evaluate the effect of morphine on mitochondrial damage and apoptosis, cytochrome c, apoptosis-inducing factor (AIF), caspase-9, and caspase-3, Bax, and Bcl-2 levels were determined from tissue samples by ELISA and immunohistochemical (IHC) methods. For in vitro analysis, CPZ C6 glioma cells were treated for 1 h, and then neurotoxic morphine (4 mM) was added to the cell medium. Cell viability was measured by the XTT method. Biochemical methods and immunofluorescence staining were used to evaluate mitochondrial damage and apoptosis. The findings indicated that co-administration of CPZ with morphine significantly reduced morphine tolerance (*p* < 0.05). Furthermore, in vivo and in vitro tests showed that CPZ administration decreased the levels of mitochondrial markers cytochrome c and AIF and proapoptotic markers caspase-3, caspase-9, and Bax and significantly increased the expression of antiapoptotic Bcl-2 (*p* < 0.01). In conclusion, both in vivo and in vitro test findings demonstrated that CPZ ameliorated morphine-induced mitochondrial dysfunction and attenuated apoptosis, reducing morphine-induced toxicity and tolerance.

**Graphical Abstract:**

Possible mechanism of the morphine tolerance reducing effect of TRPV1 channel inhibition by capsazepine. Chronic morphine administration stimulates Ca^2+^ secretion through TRPV1 channel by the activation of MOR. Increased Ca^2+^ passes into mitochondria, and excessive accumulated Ca^2+^ causes mitochondrial dysfunction and activates apoptotic mechanisms. Neuronal apoptosis results in morphine tolerance. CPZ attenuates morphine tolerance by inhibiting TRPV1 channel. MOR, mu opioid receptor; TRPV1, transient receptor potential cation channel 1; AIF, apoptosis-inducing factor; Cyt-c, cytochrome c

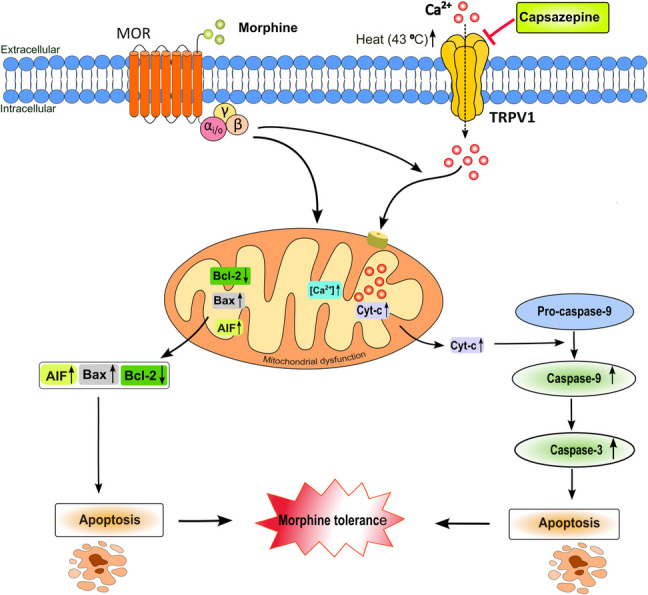

**Supplementary Information:**

The online version contains supplementary material available at 10.1007/s00210-025-04384-5.

## Introduction

Opioid drugs such as morphine are powerful pharmaceutical agents frequently used in the treatment of chronic and severe pain in the clinic (Ozdemir et al. 2011; Yang et al. 2023). Opioid drugs, like endogenous opioids, show their analgesic effects by binding to opioid receptors located on the presynaptic terminals of nociceptive primary afferent neurons (Dirik et al. 2024). Chronic administration of morphine can activate neurodegenerative, neuro-inflammatory, and apoptotic pathways and cause morphine dependence and tolerance (Motaghinejad et al. 2014; 2015a; 2016). One of the most important problems in morphine use is the development of tolerance to its antinociceptive effect with long-term use (Baser et al. 2021). Several mechanisms have been identified that contribute to the cellular processes underlying opioid tolerance. These mechanisms include downregulation of mu-opioid receptors (MOR), oxidative stress, apoptosis, microglial activation, and serotoninergic and nitric oxide pathway dysfunction (Hassanzadeh et al. 2011; Zhang et al. 2024; Ciltas et al. 2025). However, the mechanisms responsible for morphine tolerance have not yet been fully elucidated.

One of the important features of morphine-induced hyperalgesia is the increased response to thermal stimuli. Some evidence suggests that transient receptor potential vanilloid type 1 (TRPV1) channels may be an important factor in the generation of this response (Bao et al. 2015). TRPV1 is a nonselective cation (Ca^2+^) channel that is stimulated by multiple factors including noxious heat (> 42 C), acidic pH, endogenous lipids, and capsaicin (Bao et al. 2015; Por et al. 2012; Tominaga and Iwata 2025). TRPV1 is specifically expressed in the dorsal horn of the spinal cord and DRG and plays an important role in pain transmission. However, TRPV1 antagonists show dose-dependent thermal hyperalgesia reversal activity (Chahl 2024). Although TRPV1 antagonists have been shown to reduce acute and neuropathic pain, their role in the analgesic effect and tolerance of morphine has not been fully elucidated (Mehrabadi et al. 2020). The co-localization of TRPV1 channels and MOR receptors in the spinal cord and DRG neurons facilitates their interaction (Chen et al. 2008). Studies show that there are important interactions between TRPV1 channels and MOR and that morphine increases TRPV1 channel activity (Endres-Becker et al. 2007). Chronic morphine administration has been reported to increase TRPV1 expression in the DRG, spinal cord, and sciatic nerve (Chen et al. 2008; Vardanyan et al. 2009). In addition, increased TRPV1 receptor expression in the DRG is seen in morphine-resistant cancer pain (Niiyama et al. 2007). Opioid agonists and TRPV1 receptor agonists cause opposing effects. Capsaicin treatment, a TRPV1 agonist, inhibits the antinociceptive effects of morphine in rats (Scherer et al. 2017).

One of the important mechanisms underlying the development of tolerance to morphine is mitochondrial oxidative stress, neurotoxicity, and apoptosis (Ciltas et al. 2025; Osmanlıoglu et al. 2020). However, neurotoxicity and apoptosis can be observed in many conditions that cause excessive activation of TRPV1 channels (Chen et al. 2024; Uslusoy et al. 2017). Prolonged activation of TRPV1 channels leads to intracellular Ca^2^⁺ accumulation and mitochondrial Ca^2^⁺ overload in sensory neurons. This overload triggers mitochondrial depolarization, which facilitates the release of cytochrome c through the permeability transition pore (Juárez-Contreras et al. 2020). Cytochrome c activates caspase-3 through the activation of caspase-9, causing cell death. As a result, excessive activation of TRPV1 receptors leads to mitochondrial dysfunction, making neuronal cell death inevitable (Awad-Igbaria et al. 2025). Increased mitochondrial Ca^2^⁺ levels are associated with prolonged presynaptic activity, sustained glutamate release, and prolonged postsynaptic activation (Kievit et al. 2022).

In this context, it may be possible to increase the antinociceptive effects of morphine by blocking TRPV1 channels. Capsazepine (CPZ) is a TRPV1 channel antagonist and has been reported to enhance morphine-induced antinociceptive effects in mice (Nguyen et al. 2010). Therefore, this study aimed to investigate the effects of the TRPV1 antagonist CPZ on morphine-induced mitochondrial damage, apoptosis, morphine tolerance, and morphine neurotoxicity in C6 cells in rats in vivo and in vitro.

## Material and methods

### In vivo experiment

In the study, 48 adult male Wistar Albino rats, 4 months old (230–250 g), were used and kept in standard cages. Animals were obtained from the Local Experimental Animal Center (Cumhuriyet University, Sivas, Turkey). Housing and treatment of the rats were done according to the “Guide for the Care and Use of Laboratory Rats” (Laboratory Animal Resources Institute, Life Sciences Commission, 2011). Rats were housed 4–5 per cage under pathogen-free conditions in the animal care center for 7 days to acclimate. The room temperature (22 ± 5 °C), humidity 52–54%, and light/dark cycle were adjusted to 12:12 h (lights on at 7:00 AM). Standard laboratory chow and tap water were available ad libitum. Experiments were performed between 09:00 and 17:00 by researchers who were blinded to the conditions. Before the study, approval was obtained from the Cumhuriyet University Animal Ethics Committee for the experimental protocols (ethics no: 2022/523).

### Drugs

To investigate its antinociceptic activity, a selective TRPV1 channel synthetic antagonist CPZ (Sigma-Aldrich, St. Louis, MO, USA) was administered intraperitoneally (i.p.) at a dose of 3 mg/kg (Heymann et al. 2017). A single dose of morphine HCl (5 mg/kg) (Galen Medicine, Istanbul, Turkey) was administered subcutaneously (s.c.). To induce morphine tolerance in rats, 10 mg/kg morphine was injected twice daily (Wu et al. 2021). Dimethyl sulfoxide (DMSO) was used to dissolve the drug CPZ, and a 0.9% NaCl solution was used for morphine HCl. After weighing the animals, 0.1 ml of drug per 100 g of body weight was administered to each rat. Previous studies with similar designs were reviewed to determine appropriate drug doses (Hasanein and Shakeri 2014).

#### Experimental protocol

**Fig. 1:**
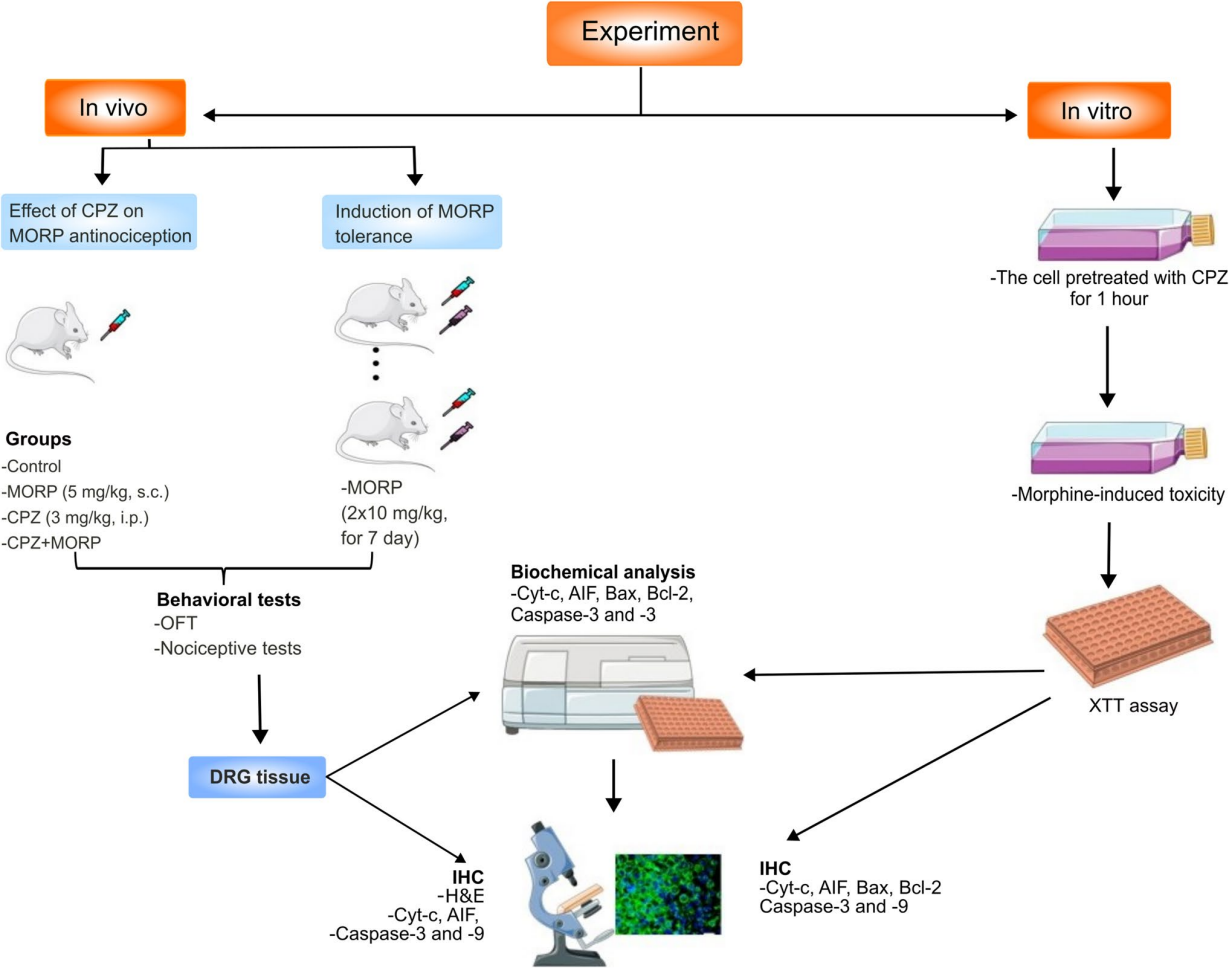
Timeline diagram representing in vivo and in vitro experimental design. CPZ, capsazepine; MORP, morphine; IHC, immunohistochemistry; OFT, open field test; TF, tail-flick test; HT, hot-plate test

Experimental animals were randomly divided into six groups: control, CPZ, morphine, CPZ + morphine, morphine-tolerant, and CPZ + morphine-tolerant (*n* = 6). To evaluate the analgesic effect of CPZ, a single dose of CPZ (3 mg/kg i.p.) and morphine (5 mg/kg s.c.) was administered (Fig. 1). To investigate the effects of CPZ on morphine tolerance, CPZ was injected twice daily for 7 days, and morphine was administered at a dose of 10 mg/kg (s.c.) 30 min later. In addition, morphine 10 mg/kg was injected twice daily for 7 days to induce morphine tolerance. Following the nociceptive behavior test measurement of each animal in the experimental groups, DRG was collected for biochemical and immunohistochemical (IHC) analyses. In the IHC-score analysis, microscopic findings obtained by immunohistochemical method were evaluated. In vitro experiment used XTT test to evaluate the effect of CPZ on morphine neurotoxicity and cell viability. Then, Cyt-c, AIF, Bax, Bcl-2, caspase-3, and caspase-9 levels were measured from glial cell homogenates by biochemical analysis, IHC, and immunofluorescence staining.

### Induction of tolerance to morphine

To induce tolerance to morphine, rats were injected with 10 mg/kg morphine HCl twice daily at 09:00 and 17:00 for 7 days (Ozsoy et al. 2023). On the 8th day of the experiment, morphine was administered at a trial dose (5 mg/kg), and morphine tolerance was analysed with analgesia tests (Tail-flick and Hot-plate tests). Analgesia tests were performed at 30-min intervals and at 0, 30, 60, 90, and 120 min after drug administration.

### Tail-flick test

The tail flick test (May TF 0703 Tail Flick Unit, Commat, Turkey) was performed by placing the stimulus 3 cm in front of the tail tips of rats to assess the activity of the spinal reflex arc. The duration of the tail flick was then recorded in seconds (Kanaan et al. 1996). The infrared intensity was set to a baseline tail flick latency (TFL) of 2.8 ± 0.4 s. Rats with baseline TFL values below 2.4 s or above 3.2 s were excluded from experimental testing. The test cut-off latency was set at 15 s to reduce the risk of tissue injury (Ozdemir et al. 2023).

### Hot plate test

The hot plate test (Commat, May AHP 0603 Analgesic Hot Plate) consists of a transparent Plexiglas cylinder with a diameter of 15 cm and a height of 22.5 cm and a base surface with adjustable temperature. The reflex response time of each animal in the experiment to the plate surface temperature of 55 ± 0.5 °C is recorded in seconds. In this experiment, pain transmission was assessed by recording the time from the moment the animal was placed on the plate until a noticeable pain response, such as rapidly withdrawing its foot, licking, or contracting its limbs. The test cut-off time for this test was set as 30 s to prevent any damage to the feet of the rats (Hassanzadeh et al. 2011). In the experiments, the pain threshold values of the rats were tested before the injection, and rats with abnormal threshold values were excluded from the test. In addition, to minimize external effects and researcher bias, nociceptive tests were performed simultaneously in a quiet room by the same researchers, unaware of the randomization. Furthermore, a two-day gap was left between each test to prevent the animals from suffering too much.

### Open field test

The open field test (OFT) was performed in a quiet, evenly lit room with low light intensity. This test was performed to analyze whether the locomotor movements of the animals were normal before the nociceptive tests. The open field apparatus consisted of 16 squares, each measuring 25 × 25 cm, separated by white lines on a black background. The edges of the apparatus were surrounded by a black wooden wall with a height of 30 cm. During the experiment, each animal was subjected to the open field test 60 min after drug injection. The animals were allowed to explore the new environment for 2–3 min to get used to the open field until they felt comfortable in the new environment (Ghafarimoghadam et al. 2022). After the animals were individually placed in the center of the apparatus, their behavior was automatically recorded by a computerized video monitoring system (Smart 3.0, Panlab, Barcelona, ​​Spain) for 5 min. To minimize possible effects from previous tests, the apparatus was cleaned using a 50% ethanol solution after each session. From the records obtained in the open field test, the number of squares covered by the animals, vertical activity, and grooming was analyzed in order (Cetin et al. 2024).

### Data analysis

To evaluate analgesic efficacy, the animals’ paw and tail withdrawal latencies in antinociceptive tests were calculated in seconds, and the following formula was used to determine the maximum possible effect (% MPE):

% MPE = [(test latency-baseline)/(cutoff-baseline)] × 100.

The average of the % MPE values in each group was taken for statistical analysis.

### DRG tissue ısolation

After completion of the test measurements, rats were sacrificed by cervical dislocation and DRGs were isolated from the L4-S5 level of the spine. DRG tissues were collected in petri dishes and then washed three times with cold phosphate-buffered saline (PBS) (pH 7.4). The samples were then mixed thoroughly in cold PBS using a mechanical homogenizer (SpeedMill PLUS; Jena, Germany). They were then centrifuged at 4000 rpm for 10 min at 4 °C. The supernatants were used for biochemical analyses. Total protein levels were measured using the Bradford protein assay kit (SERVA, Heidelberg, Germany).

### Determination of cytochrome-c, AIF, Bcl-2, Bax, caspase-9, and caspase-3 levels

Commercial rat-specific ELISA kits (Laboratory of Bioassay Technology, Shanghai, China) were used to measure the levels of cytochrome c (cat no: E1240Ra), AIF (apoptosis-inducing factor) (cat no: E0215Ra), Bcl-2 (cat no: E0037Ra), Bax (cat no: E1869Ra), caspase-9 (cat no: E1898Ra), and caspase-3 (cat no: E1648Ra) in the collected DRG supernatants (Singh et al. 2024). The standard kit and tissue samples were plated according to the manufacturer’s instructions. After completion of the assay, the absorbance values ​​of each well were determined by reading at a wavelength of 450 nm. Standard curves were constructed for quantification of the samples. The variation in the coefficients observed both within and between plates was less than 10%, indicating that the results were highly sensitive and reproducible.

### Histopathological analysis

Samples prepared from rat DRG tissues were placed in 10% neutral formalin solution. Tissues were subjected to routine alcohol-xylene dehydration and placed in paraffin blocks. Then, 4 µm sections were taken from DRGs and transferred to slides. Tissues were stained with H&E and evaluated for necrotic-degenerative changes, mononuclear cell infiltration, and hemorrhagic changes.

### Immunohistochemical (IHC) method

A section of DRGs obtained from rats was treated with 10% neutral formalin solution for histopathological analysis. The tissues were subjected to routine alcohol-xylene dehydration and embedded in paraffin blocks. Then, 4 µm sections were cut from DRGs and mounted on slides. Sections on polylysine slides were subjected to a series of treatments including xylene and alcohol and washed first with ultrapure water and then with PBS for 15 min. Sections were trypsinized (2% trypsin in 50 mM Tris buffer) for 20 min at 37 °C. Then, circles were drawn around the tissues on the slide with a hydrophobic barrier pen, and the tissues were subjected to inhibition of endogenous peroxidase activity with 3% H2O2 for 10 min. To identify antigen, the tissues were exposed to an antigen retrieval solution at 500 watts twice for 5 min. In the next step, they were incubated with primary antibodies of cytochrome-c (Abcam, Cambridge, UK), AIF (Bioss, MA, USA), Bcl-2 (Bioss, MA, USA), Bax (Bioss, MA, USA), caspase-9 (Bioss, MA, USA), and cleaved caspase-3 (Elabsceience, Houston, USA) overnight at + 4ºC. The secondary reagent used was HRP (Thermo Fischer, cat no: TP-125-HL). 3,3′-Diaminobenzidine (DAB) was used as chromogen. After application of Mayer’s hematoxylin counterstain, the slides were covered with Entellan and evaluated using a light microscope (Olympus BX-51, Olympus, Japan). Following staining, at least five views from each section were used to quantitatively determine mitochondrial damage markers and proapoptotic/antiapoptotic proteins by measuring relative primary antibody expression using ImageJ software version 1.50 (National Institutes of Health, Bethesda, Maryland, USA).

#### In vitro experiment

### Cell culture

In this study, C6 Glioma (CRL107) cell lines, which are known to elicit neurotoxic responses to morphine, were used (Zhou et al. 2011). C6 glial cell lines were provided by the American Type Culture Collection (ATCC). The culture medium used for cell growth consisted of Dulbecco’s Modified Eagle Medium (DMEM) enriched with 10% FBS, 1% L-glutamine, and 1% penicillin/streptomycin (Thermo Fisher Scientific, Altrincham, UK). The cells to be used in the experiment were kept in a humidified environment with 5% CO2 at 37 °C.

### Cell viability

The XTT test (Roche Diagnostic, MA, USA) was used to evaluate the effect of morphine and CPZ on cell viability. Conversion of tetrazolium salt to formazan was used as the criterion to evaluate cell viability by following the manufacturer’s instructions for the XTT assay kit. Cells were plated in 96-well plates (approximately 10,000 cells/well) and cultured for 24 h. They were then treated with morphine and various concentrations of CPZ for an additional 24 h. After the incubation period, cells were removed and each well went through two PBS washes. In the final step, 100 μL of DMEM without phenol red and 50 μL of XTT mix solution were added to each well. The plates were then incubated at 37 °C for 4 h. An ELISA microplate reader was used to quantitate the plates at 450 nm. The live cell ratio was determined relative to the control group, and all experiments were repeated three times.

### Morphine-induced neurotoxicity analysis

To evaluate the effects of CPZ on morphine cytotoxicity, cells were divided into four groups. First, glial cells were treated with different doses of morphine (1.5, 2, 3, 4, 6, 8, 12, and 16 mM) to determine the 50% lethal dose of morphine. Then, lethal dose (4 mM) of morphine was applied to glial cells for 24 h, and CPZ was added to the same cells at different concentrations (1.25, 2.5, 5, 10, and 20 µM) to examine the effects on cell viability.

### Preparation of cell homogenates

Cells from each group were collected and placed in sterile tubes. Then, a 10-min centrifugation at 2000 rpm was performed to remove the supernatants. The cells in the tubes were suspended in PBS (pH 7.4) at a density of approximately one million per millilitre. The cells were subjected to a series of freeze–thaw cycles to allow the internal contents to escape. The tubes were centrifuged at 4000 rpm and 4 °C for 10 min. The supernatants were then collected and subjected to a biochemical examination. Total protein concentrations were determined using the Bradford protein assay kit (SERVA, Heidelberg, Germany).

### Immunofluorescence staining

For immunofluorescence staining, cells were first fixed in methanol at − 20 °C for 5 min and then washed with PBS. Samples were incubated in PBS supplemented with 0.1% Triton X-100 at ambient temperature for 15 min. In the next step, cells were rinsed and incubated in PBS containing 2% BSA for 60 min. Following incubation, samples were treated with primary antibodies Bax, Bcl-2 (bs-0032R, Bioss, Beijing, China), cytochrome c (Abcam Limited, Cambridge, UK), AIF, caspase-9 (bs-0049R, Bioss, Beijing, China), and cleaved caspase-3 (E-AB-30004, Elabscience, Houston, TX, USA) at 1/200 dilution at + 4 °C overnight. The PBS washed cells were incubated with goat anti-mouse FITC ((Jackson ImmunoResearch Lab. West Grove, Pennsylvania, USA) and goat anti-rabbit FITC (Jackson ImmunoResearch, West Grove, PA) secondary antibodies at a dilution of 1/50 for 1 h at room temperature in the dark. After the cells were washed, 4′,6-diamidino-2-phenylindole (DAPI, D9542, Sigma-Aldrich) was added, and the prepared slides were examined using a Zeiss Axio fluorescence microscope (Zeiss Axio Scope A1, Zeiss, Germany). Fluorescence intensity was analyzed in six random fields with Fiji ImageJ software (ImageJ software version 1.50, Bethesda, MD, USA).

### Statistically analysis

Data obtained from each group in the study were expressed as mean ± S.E.M. Before statistical analysis, the normality of the distribution of the data was assessed using the Shapiro–Wilk test. Since the test results showed normal distribution, parametric tests were used in the analysis of the data. Since there were more than two groups, one-way and repeated measures analysis of variance (ANOVA) was used in the analysis of data, and multiple comparisons were performed with the Tukey post hoc test (SPSS computer program, version 23.0 for Chicago, IL, USA). *p* < 0.05 was considered statistically significant in all groups.

## Results

### Effects of CPZ on morphine analgesia and tolerance

**Fig. 2:**
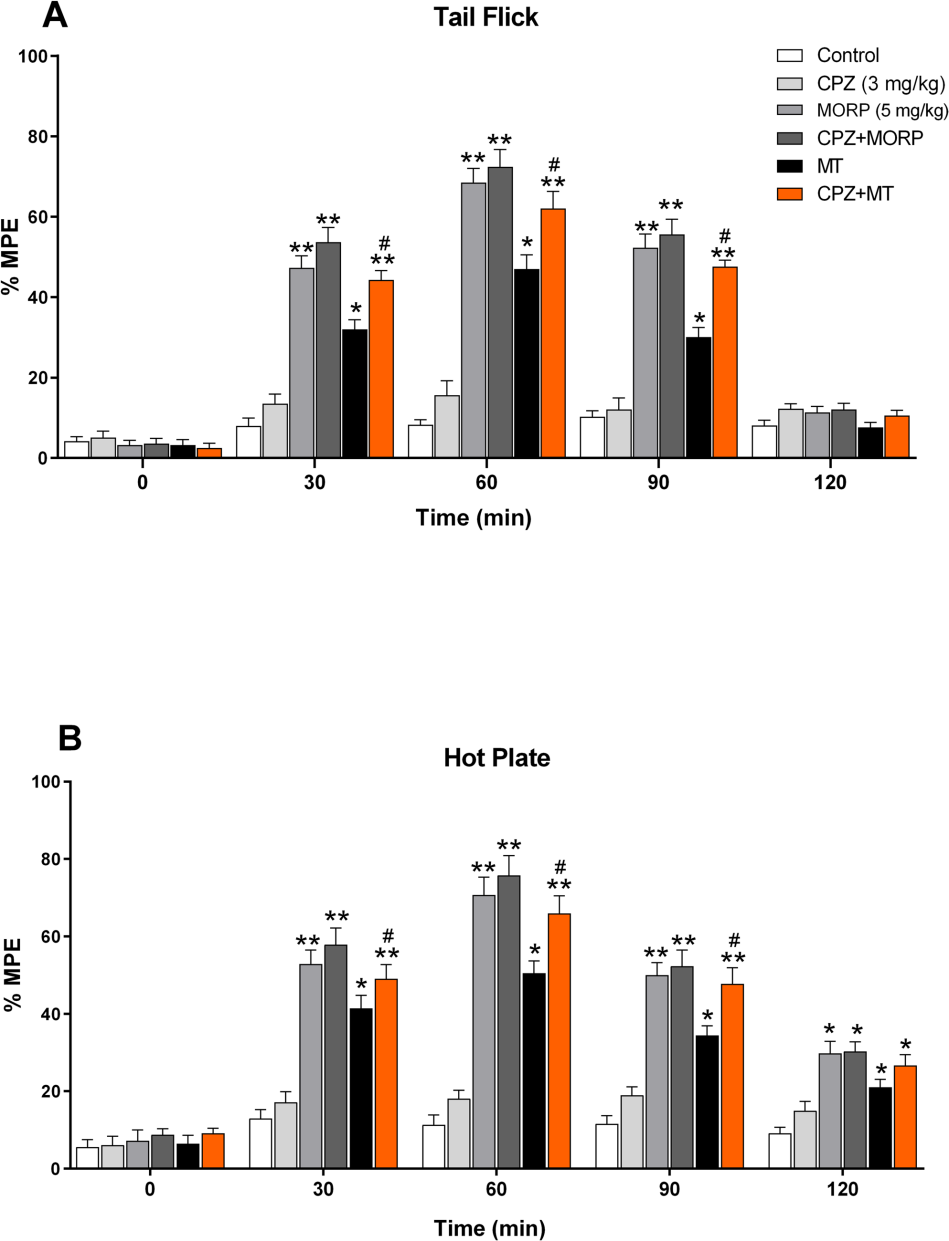
Effects of CPZ on morphine analgesia and tolerance. The analgesic effect (% MPE) of CPZ + MT group was significantly higher in both tail-flick (**A**) and hot-plate test (**B**) compared to the MT group (*p* < 0.05). Each point represents the mean ± SEM of % MPE for 6 rats. *p < 0.05, **p < 0.01 vs. control and ^#^*p* < 0.05 vs. MT group (one-way ANOVA followed by Tukey HSD post hoc test). %MPE, Percent maximum possible effect; CPZ, capsazepine; MT, morphine tolerant

The results of antinociceptive tests (tail-flick and hot-plate tests) showed that the antinociceptive effect in rats administered CPZ (3 mg/kg) was not statistically significant when compared to the control group (*p* > 0.05). Repeated measurements in the morphine-tolerant group showed that the antinociceptive effect was significantly decreased compared to the morphine group (*p* < 0.05; *F*_5,24_ = 13.25). However, the antinociceptive effect that was decreased in the morphine-tolerant group was significantly increased with concurrent CPZ in both tail-flick (Fig. 2A; *p* < 0.05) and hot-plate tests (Fig. 2B; *p* < 0.05) compared to the morphine-tolerant group.

### Effect of CPZ on motor activity

**Fig. 3:**
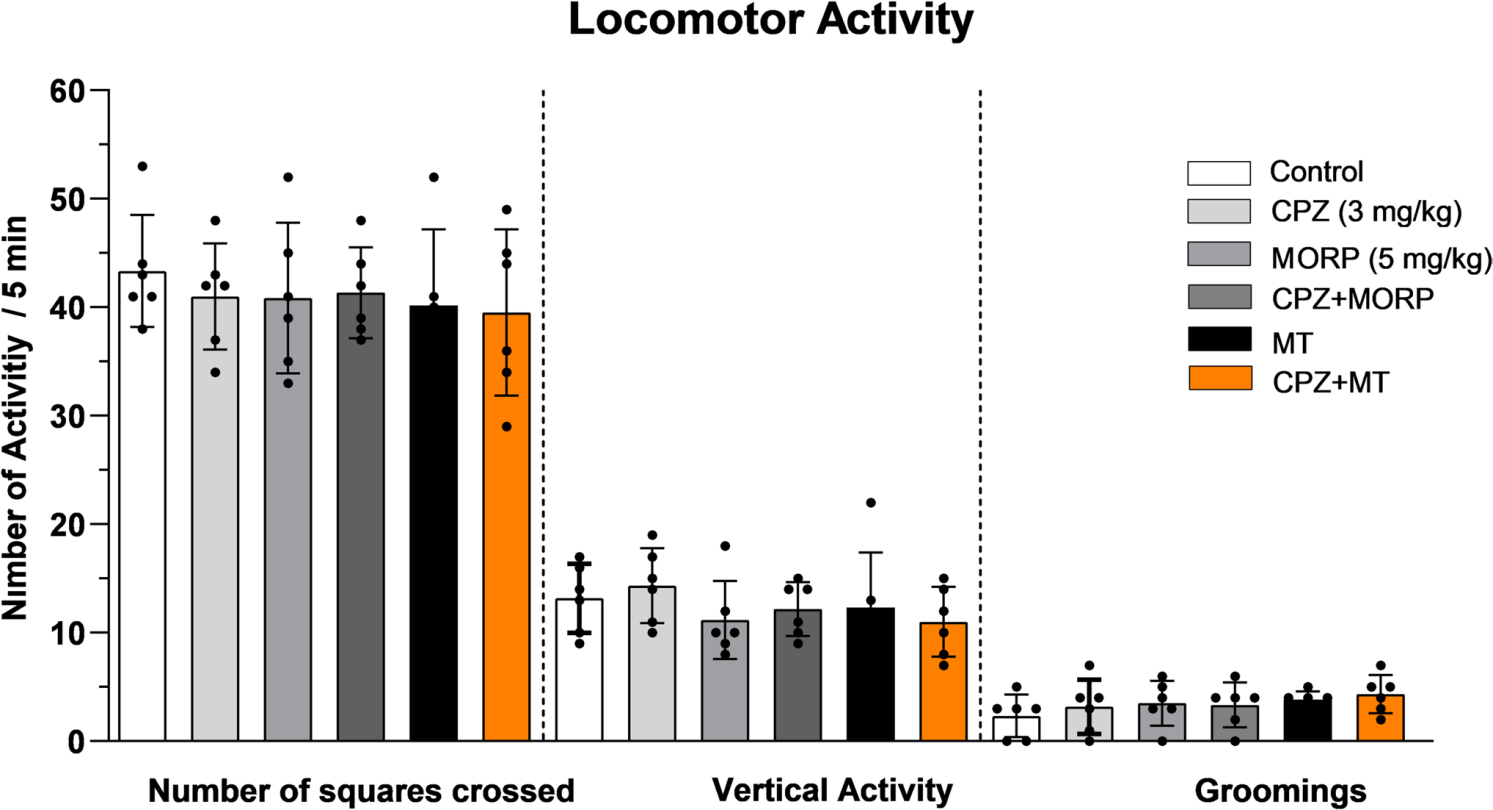
Effects of CPZ and morphine on locomotor activity. The values are presented as means ± SEM (*n* = 6)

The recordings obtained from the rats in the open field test were analyzed in terms of the number of frames they covered, vertical activity, and grooming frequency measurements, and it was determined that there was no statistically significant difference between the groups (*p* > 0.05, Fig. 3).

#### Effect of CPZ on cytochrome c, AIF, caspase-3, caspase-9, Bax, and Bcl-2 expressions in DRG

**Fig. 4:**
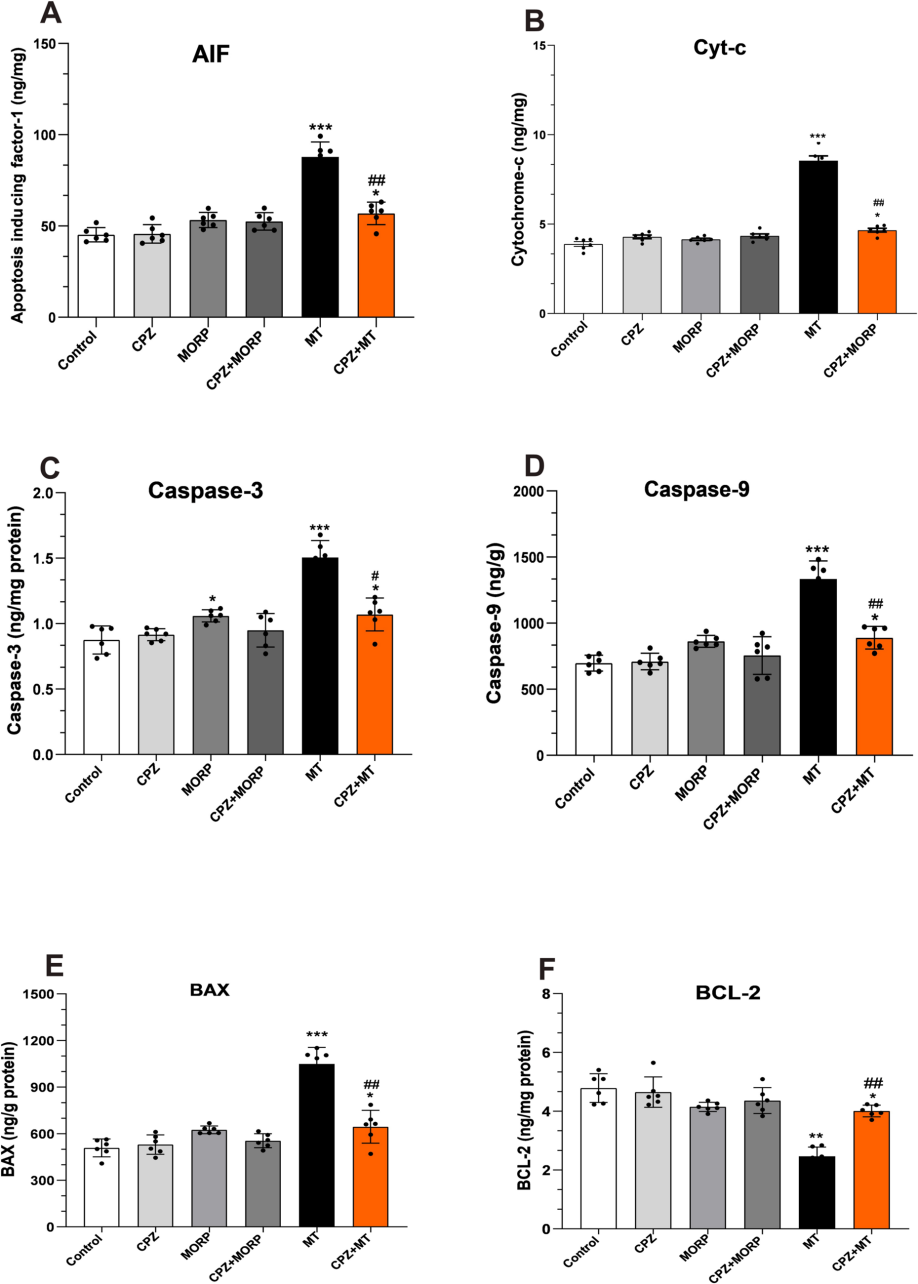
Effect of CPZ on AIF (**A**), cytochrome c (**B**), caspase-3 (**C**), caspase-9 (**D**), Bax (**E**), and Bcl-2 (**F**) expressions in DRG. Values are presented as mean ± SEM for 6 rats. *p < 0.05, **p < 0.01, and ***p < 0.001 vs. control and ^#^*p* < 0.05, ^##^*p* < 0.01 vs. MT group (one-way ANOVA followed by Tukey HSD post hoc test). CPZ, capsazepine; MT, morphine toleran

Statistical test results showed that both cytochrome c and AIF expressions in DRG tissue were significantly increased in the morphine-tolerant group (*p* < 0.001; *F*_5,30_ = 18,35, Fig. 4A and B). However, administration of CPZ together with morphine significantly decreased the expressions of cytochrome c and AIF (*p* < 0.001). Proapoptotic markers caspase-3, caspase-9, and Bax levels were significantly increased in DRG tissue in morphine-tolerant rats compared to control (*p* < 0.001, Fig. 4C, D, and E). However, administration of CPZ together with morphine to rats caused a statistically significant decrease in the levels of these proapoptotic markers (*p* < 0.001). On the other hand, the expressions of Bcl-2, an antiapoptotic protein, were significantly decreased in the morphine-tolerant group compared to control (*p* < 0.01, Fig. 4F). However, the addition of CPZ to morphine-tolerant rats significantly increased Bcl-2 expression in DRG (*p* < 0.01).

### Effect of CPZ on histopathological changes

**Fig. 5:**
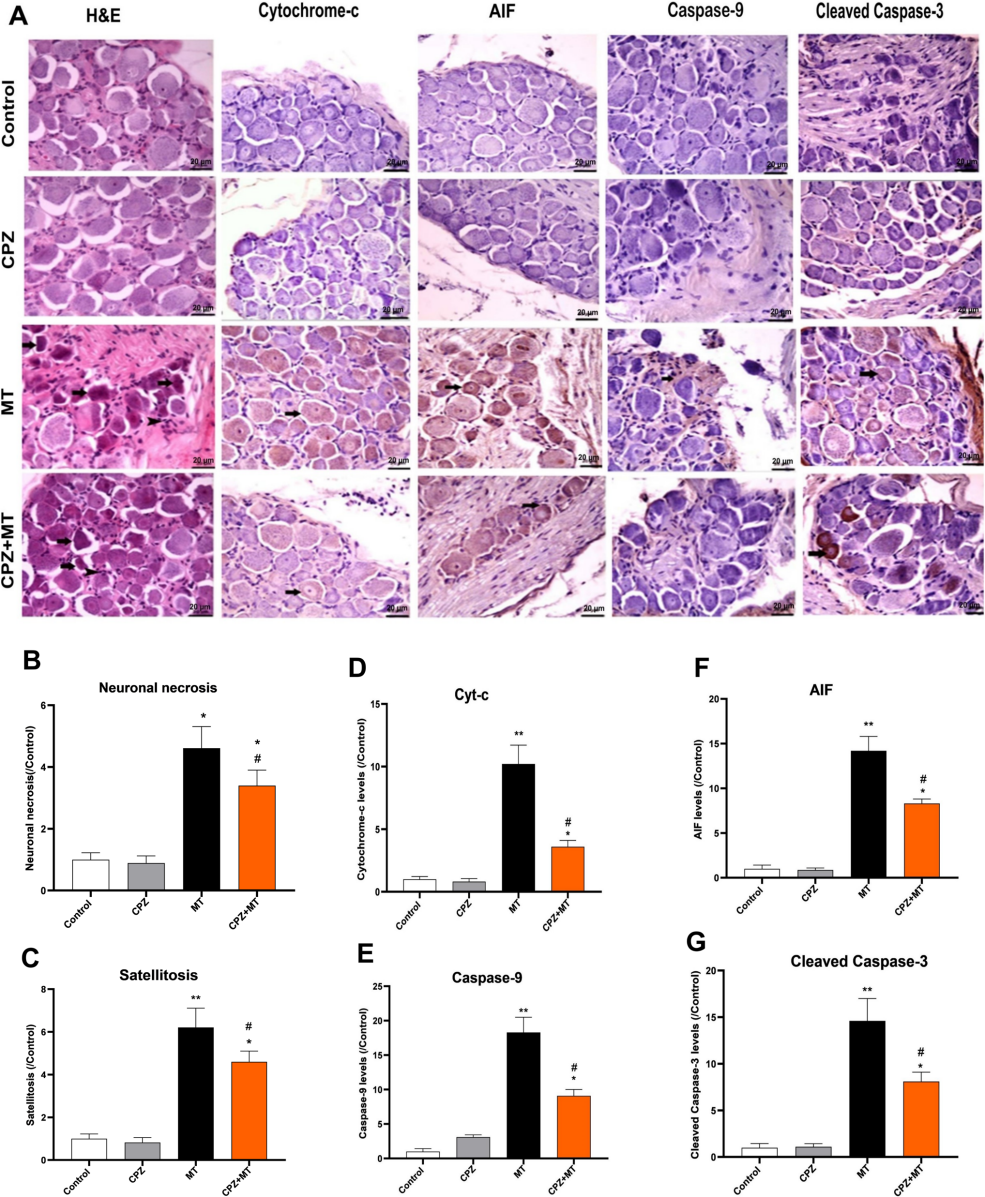
Effect of capsazepine on neuronal necrosis, satellitosis, Cyt-c, AIF, caspase-9, and cleaved caspase-3 levels in DRG. **A** H&E and IHC stainings in DRG slides, and **B**, **C**, **D**, and **E** quantitative IHC analysis of Cyt-c, AIF, caspase-9, and caspase-3, respectively. (Magnification × 200). Values are presented as mean ± SEM for 6 rats. *p < 0.05 and **p < 0.01, vs. control and ^#^*p* < 0.05, vs. MT group (one-way ANOVA followed by Tukey HSD post hoc test). Black arrows indicate necrotic and satellite cells. Scale bar, 20 μm

Histopathological findings showed increased levels of necrosis and satellite disease in the morphine-tolerant group rats compared to the control group, and hemorrhagic areas were present in some places. However, the addition of CPZ to the morphine-tolerant group rats significantly reduced the rates of necrosis and satellite disease (Fig. 5A).

### Effect of CPZ on cytochrome c, AIF, cleaved caspase-3, caspase-9, Bax, and Bcl-2 expressions in IHC analysis

IHC analysis showed that the expression levels of mitochondrial damage markers cytochrome c and AIF were statistically significantly increased in morphine-tolerant rats compared to the control group (*p* < 0.01; *F*_3,15_ = 17.36, Fig. 5B, C). However, concomitant administration of CPZ to morphine-tolerant rats significantly decreased the expressions of cytochrome c and AIF (*p* < 0.05). While the expression of proapoptotic proteins caspase-9 and cleaved caspase-3 was significantly increased in morphine-tolerant rats compared to the control group (*p* < 0.01, Fig. 5D and E), administration of CPZ to morphine-tolerant rats caused a significant decrease in the expression of caspase-9 and cleaved caspase-3.

### Effect of CPZ on morphine-induced neurotoxicity in C6 cells

**Fig. 6:**
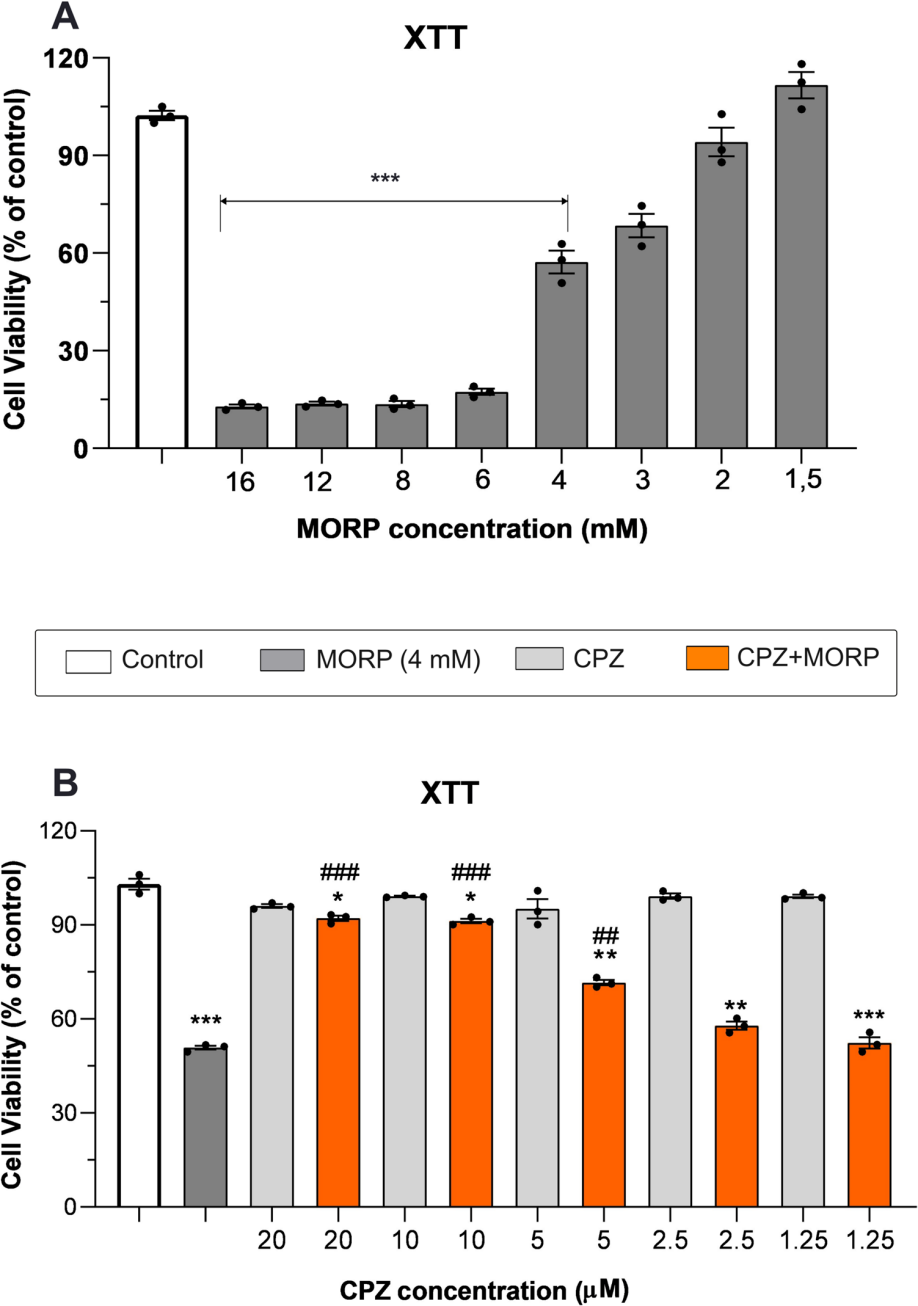
The data illustrate the impact of CPZ at varying concentrations on cell viability following morphine-induced cytotoxicity in C6 cells. **A** The C6 cells were exposed to morphine at various concentrations (1.5, 2, 3, 4, 6, 8, 12, and 16 mM) for a duration of 24 h. C6 cells were treated with CPZ at various concentrations (20, 10, 5, 2.5, and 1.25 µM) in combination with 4 mM morphine. Data are represented as mean ± SEM. *p < 0.05, **p < 0.01, ***p < 0.001 vs. control group and ^##^*p* < 0.01, ^###^*p* < 0.001 vs. MORP group. MORP, morphine; CPZ, capsazepine

The results of the XTT test applied to evaluate the neurotoxicity of morphine demonstrated that 50% lethal concentration of morphine was 4 mM (Fig. 6A). Morphine applied at a dose of 4 mM significantly decreased cell viability compared to the control (*p* < 0.001; *F*_3,15_ = 21,3). The decrease in cell viability intensified with increasing morphine dose. To investigate the protective role of CPZ against morphine-induced neurotoxicity, cells were treated with different concentrations of CPZ (20, 10, 5, 2.5, and 1.25 µM) 1 h before morphine administration. CPZ administered at concentrations of 5 µM (*p* < 0.01, Fig. 6B), 10 µM (*p* < 0.001), and 20 µM (*p* < 0.001) significantly reduced morphine-induced toxicity. Additionally, compared with untreated control cells, CPZ alone did not affect the viability of C6 cells at any concentration (*p* > 0.05, Fig. 6B).

### Effects of CPZ on cytochrome c, AIF, caspase-9, and caspase-3 protein expressions in C6 cells

**Fig. 7:**
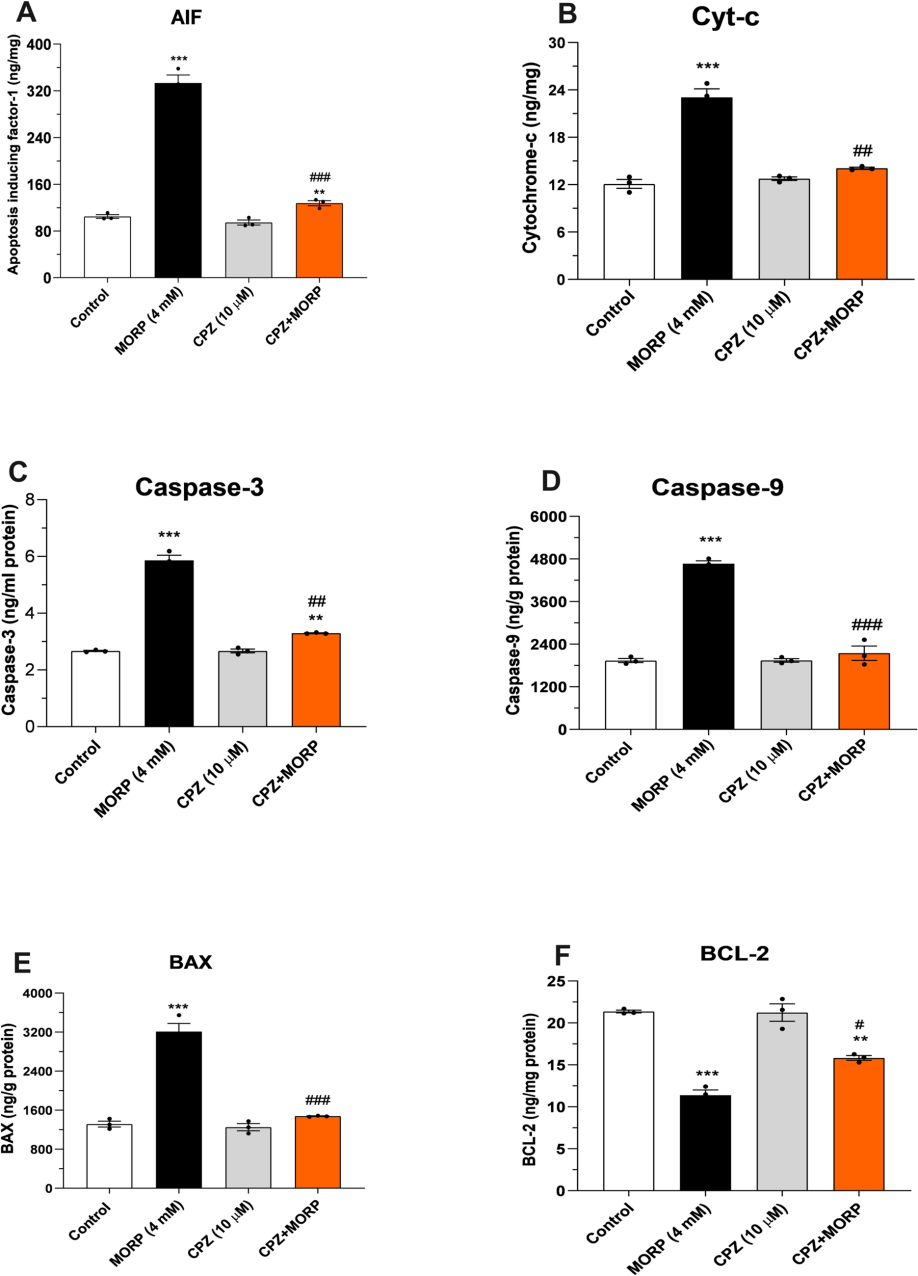
Effect of CPZ on AIF (**A**), cytochrome c (**B**), caspase-3 (**C**), caspase-9 (**D**), Bax (**E**), and Bcl-2 (**F**) expressions in C6 cells. Values are presented as mean ± SEM (*n* = 3). ***p < 0.001 vs. control and ^#^*p* < 0.05, ^##^*p* < 0.01 vs. MORP group (one way ANOVA followed by Tukey HSD post hoc test)

Biochemical results indicated that the expressions of mitochondrial damage markers cytochrome c and AIF in morphine (4 mM)-treated C6 cells were statistically significantly increased compared to the control group (*p* < 0.001, Fig. 7A and B). However, pre-treatment with CPZ (10 μM) for 1 h significantly decreased the expressions of cytochrome c and AIF relative to the morphine group (*p* < 0.001). Similarly, pre-incubation of C6 cells with morphine for 24 h significantly increased the pro-apoptotic markers Bax, caspase-9, and caspase-3 compared to the control (*p* < 0.001, Fig. 7C, E, and F). In contrast, CPZ (10 μM) pre-treatment significantly decreased these markers compared to morphine-treated cells (*p* < 0.001). Furthermore, while morphine decreased the levels of the anti-apoptotic protein Bcl-2 compared to the control group, CPZ pretreatment again significantly increased Bcl-2 levels (*p* < 0.001, Fig. 7D). Notably, CPZ alone (10 μM) did not cause significant changes in mitochondrial damage or apoptosis compared to the control group.

### Effect of CPZ on cytochrome c, AIF, Bcl-2, Bax, caspase-3, and caspase-9 expressions in immunofluorescence staining

**Fig. 8:**
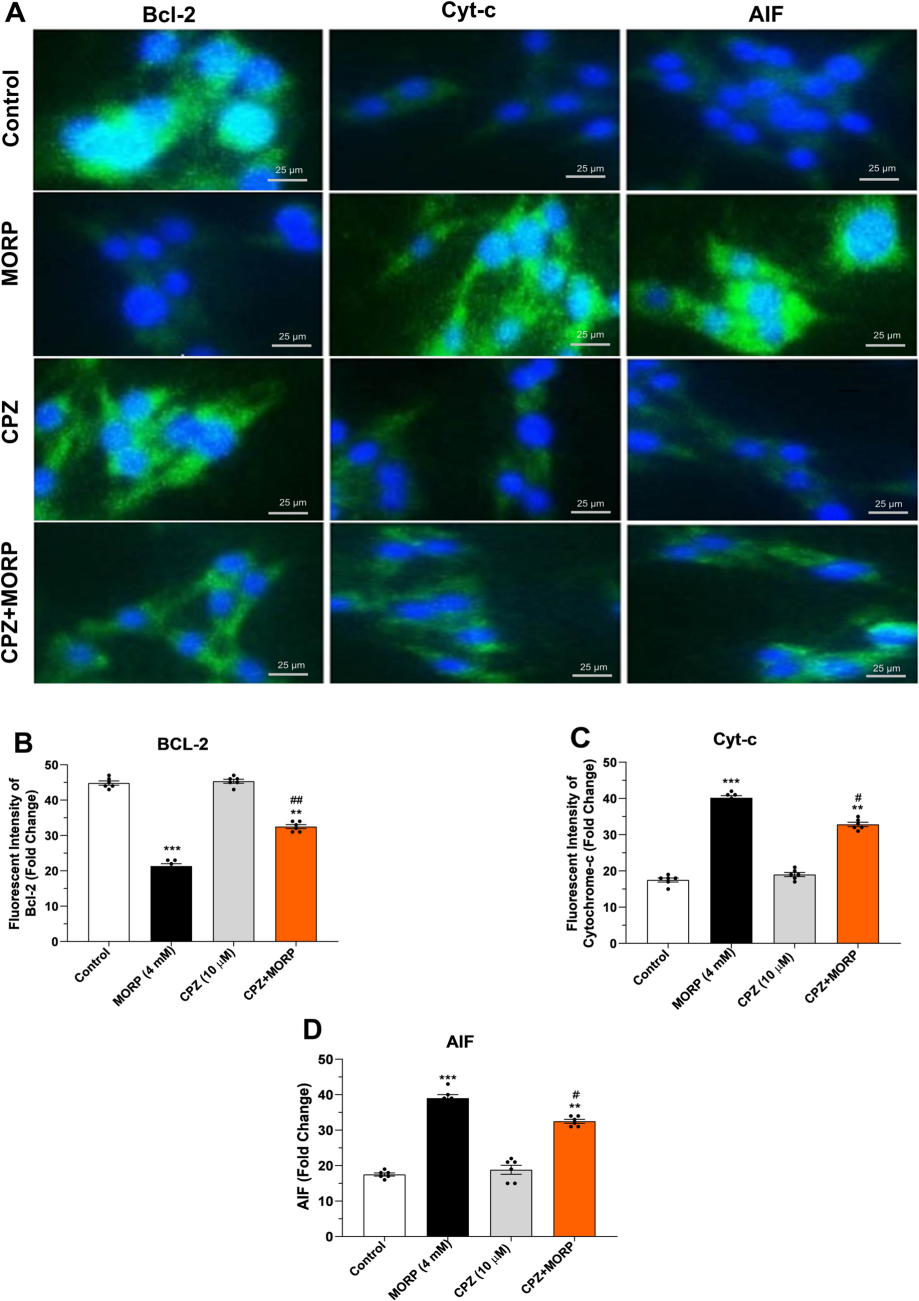
Effects of CPZ on cytochrome c, AIF, caspase-9, and caspase-3 protein expressions in C6 cells. **A** Immunofluorescence staining of cytochrome c, AIF, caspase-9, and caspase-3 protein expressions in C6 cells and **B**, **C**, and **D** quantitative analysis of Bcl-2, Cyt-c, and AIF proteins, respectively. (Magnification × 200). Values are presented as mean ± SEM (*n* = 6). **p < 0.01, ***p < 0.001 vs. control and ^#^*p* < 0.05, ^##^*p* < 0.01 vs. MORP group. Scale bar, 25 μm

**Fig. 9:**
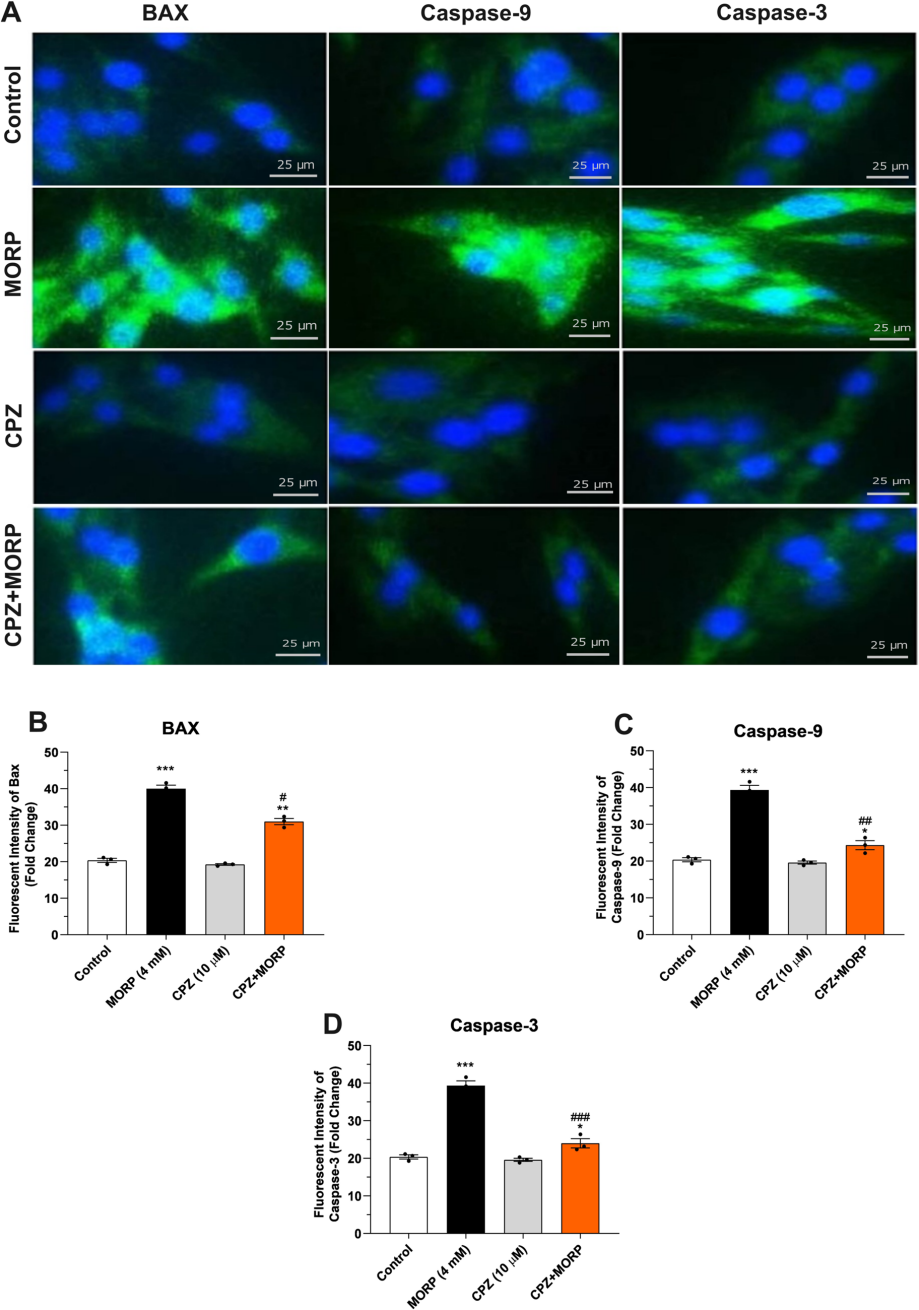
Effects of CPZ on Bax, caspase-9, and caspase-3 protein expressions in C6 cells. **A** Immunofluorescence staining of Bax, caspase-9, and caspase-3 protein expressions in C6 cells and **B**, **C**, and **D** quantitative analysis of Bcl-2, Cyt-c, and AIF proteins, respectively. (Magnification × 200). Values are presented as mean ± SEM (*n* = 6). ***p < 0.001 vs. control and ^#^*p* < 0.05, ^##^*p* < 0.01, and ^###^*p* < 0.001 vs. MORP group. Scale bar, 25 μm

Immunofluorescence staining showed that the expression of anti-apoptotic protein Bcl-2 was significantly decreased in C6 cells treated with morphine (4 mM) compared to control (*p* < 0.001, Fig. 8A and B). However, treatment with CPZ (10 μM) together with morphine significantly increased Bcl-2 expression in the cells (*p* < 0.01). Administration of morphine caused significant increases in the levels of both cytochrome c and AIF, markers of mitochondrial damage (*p* < 0.001, Fig. 8C and D). In contrast, treatment with the TRPV1 antagonist CPZ together with morphine to C6 cells caused significant decreases in the levels of both cytochrome c and AIF (*p* < 0.01). At the same time, staining for proapoptotic proteins Bax, caspase-9, and caspase-3 showed significantly increased positivity in Bax, caspase-9, and caspase-3 expressions in morphine-treated cells compared to control (*p* < 0.001, Fig. 9A, B, and C). However, application of CPZ together with morphine showed a significant decrease in Bax (*p* < 0.05), caspase-9 (*p* < 0.01), and caspase-3 (*p* < 0.01) positivity in C6 cells.

## Discussion

In this study, we investigated the potential of TRPV1 inhibitor CPZ to reduce morphine-induced tolerance in rats and prevent morphine neurotoxicity in C6 cells. As a result, antinociceptive tests demonstrated that CPZ attenuated the analgesic tolerance of morphine. Biochemical and immunohistochemical analyses revealed increased expression of cytochrome c and AIF proteins, indicators of mitochondrial damage, in DRG tissues of rats that developed morphine tolerance. In addition, elevated expressions of proapoptotic proteins such as Bax, caspase-3, and caspase-9 were observed. In contrast, administration of CPZ to rats that developed morphine tolerance reduced proteins associated with mitochondrial damage and apoptosis. These results indicated that TRPV1 channels increased mitochondrial dysfunction and apoptosis in DRG neurons and played an important role in the development of morphine tolerance. CPZ administration antagonized these effects. In addition, CPZ reduced morphine-induced neurotoxicity in C6 cells. Biochemical analysis and immunofluorescence staining showed that CPZ pretreatment decreased the levels of cytochrome c, AIF, Bax, caspase-9, and caspase-3 in C6 cells while simultaneously increasing the levels of Bcl-2 in response to morphine-induced neurotoxicity.

Accumulating evidence suggests that neuronal apoptosis (Ozdemir et al. 2023; Charkhpour et al. 2010), oxidative stress (Motaghinejad et al. 2015b), and mitochondrial damage (Xie et al. 2024) play critical roles in the development of tolerance to the antinociceptive effects of morphine. In support of this, a study reported that long-term morphine administration in rats increased the development of tolerance to morphine by increasing apoptosis (Shafie et al. 2015). In addition, it has been stated that changing the morphine dosage regimen may reduce the severity of morphine-induced neurodegeneration and apoptosis (Motaghinejad et al. 2015a). Anti-apoptotic agents released in the central nervous system alleviate opioid tolerance by inhibiting neuronal apoptosis. However, long-term morphine administration in rats induces apoptosis by causing an increase in pro-apoptotic Bax and a decrease in anti-apoptotic Bcl-2 proteins (Hassanzadeh et al. 2011; Shafie et al. 2015). Morphine increases neuroapoptosis by causing mitochondrial dysfunction and activating the apoptosis cascade Kasala et al. 2020). In addition, co-administration of calcium channel blockers with morphine has been shown to increase the acute analgesic effects of morphine and reduce morphine tolerance by reducing mitochondrial oxidative stress (Soleimanii et al. 2024).

Chronic morphine administration causes significant changes in the expression of Bax and Bcl-2 proteins and the process of apoptosis in rat models (Hassanzadeh et al. 2011; Avci et al. 2022). Studies indicate that an anti-apoptotic agent attenuates the development of opioid tolerance by increasing mRNA levels of the anti-apoptotic Bcl-2 protein while simultaneously decreasing the level of the pro-apoptotic Bax gene (Shafie et al. 2015). In vitro studies have shown that continuous exposure of neuronal cultures from certain cell lines to µ and κ opioid receptor agonists increases neuronal cell death by apoptosis (Tong et al. 2011; Mao et al. 2002). Additionally, morphine tolerance increases Bcl-2 protein expression in brain microglia cells while decreasing NF-κB, Bax, and caspase-3 protein expression (Shuai et al. 2025). In an experimental study, it was shown that opioid administration stimulated neuronal apoptosis in rat pheochromocytoma (PC12) cells by decreasing the levels of anti-apoptotic proteins while increasing the expression of pro-apoptotic Bax, caspase-3, and caspase-9 (Tian et al. 2017). Our findings from both in vivo and in vitro analyses showed that morphine administration decreased the levels of pro-apoptotic proteins, especially Bax, caspase-9, and caspase-3, while simultaneously increasing the expression of the anti-apoptotic protein Bcl-2.

Mitochondria play a critical function in regulating cellular activities, including ATP production, reactive oxygen species (ROS) production, cell cycle, and apoptosis (Babizhayev and Yegorov 2016; Su et al. 2021). Additionally, mitochondrial dysfunction may contribute significantly to morphine tolerance and dependence. Chronic intrathecal morphine administration causes an excessive increase in ROS and accumulation of damaged mitochondria in the spinal cord (Feng et al. 2013). Mitochondrial ROS produced by morphine contributes to lysosomal damage and activation of the NLRP3 inflammasome (Liu et al. 2020). Morphine induces ROS generation in neuroblastoma SH-SY5Y cells in a concentration-dependent manner, and excessive ROS contributes to morphine addiction by affecting opioid receptors with cellular damage and apoptosis (Ma et al. 2015). In the study, we showed that mitochondrial dysfunction was induced by increased expression of cytochrome c and AIF in cells exposed to morphine, and apoptosis was induced by increased expression of proapoptotic proteins caspase-3 and caspase-9 and decreased expression of anti-apoptotic protein Bcl-2. TRPV1 and MOR receptors are co-localized in DRG neurons and spinal cord, indicating possible interactions between these receptor systems (Abdullah and Altier 2020). Evidence has shown that long-term exposure to morphine leads to upregulation of TRPV1 expression in spinal cord, DRG, and sciatic nerve. In addition, the analgesic effects of morphine were enhanced in mice with bone cancer treated with the TRPV1 antagonist SB366791 (Chen et al. 2008). Our findings in thermal analgesic tests showed that morphine reduced the antinociceptive efficacy in animals that developed tolerance, whereas the administration of the TRPV1 channel antagonist CPZ increased antinociceptive efficacy and decreased tolerance. Similar to our study, Nguyen et al. (2010) showed that administering capsazepine to mice at a dose of 5 mg/kg potentiated the antinociception of morphine and prevented the development of tolerance and physical dependence to morphine. In an experimental study, it was shown that TRPV1 activity increased during opioid withdrawal due to PKA activation via cAMP (Spahn et al. 2013). Researchers suggested that this increase in TRPV1 activity is a mechanism underlying the hyperalgesia in DRG neurons due to opioid withdrawal. Similarly, the TRPV1 antagonist SB-36679 was shown to increase the analgesic efficacy of morphine in mice and reduce the development of tolerance (Mazeto et al. 2020). Thermal hyperalgesia was not observed after chronic morphine administration in TRPV1 knockout mice (Vardanyan et al. 2009).

TRPV1 channels play important roles in intracellular Ca^2^⁺ homeostasis (Juárez-Contreras et al. 2020; Xu et al. 2020). Studies in primary DRG neuronal cultures have shown that excessive application of TRPV1 agonist compounds such as capsaicin and resiniferatoxin disrupts mitochondrial Ca^2^⁺ homeostasis and causes Ca^2^⁺ accumulation (Stueber et al. 2017; Özdemir et al. 2016). In addition, experimental spinal cord injury activates TRPV1 and TRPM2 in DRG neurons, causing mitochondrial dysfunction, ROS accumulation, and apoptosis via caspase pathway activation (Özdemir et al. 2016). TRPV1 activation contributes to microglial injury via Ca^2^⁺ signaling and mitochondrial dysfunction (Kim et al. 2006). At the same time, the TRPV1 activator capsaicin causes mitochondrial dysfunction in trigeminal ganglion neurons and PC12 cells expressing the TRPV1 receptor. This results in dose-dependent mitochondrial toxicity, structural damage, and increased sensitivity to heat through mitophagy (Shibata et al. 2020). This study highlights the essential role of the TRPV1 cation channel in the development of tolerance, where its activation in DRG neurons leads to mitochondrial damage and altered apoptotic protein expression. The study results suggest that CPZ may enhance the analgesic effect of morphine by reducing excessive Ca^2^⁺ influx, thereby protecting cells from both tolerance and neurotoxicity.

The main limitation of this study is that, although ELISA and IHC methods were used to determine the levels of mitochondrial damage and apoptosis biomarkers in DRG tissue while evaluating morphine-induced neurotoxicity, the results were not confirmed by the Western blot technique. In addition, another important study limitation is that a TRPV1 channel agonist was not used as a positive control while evaluating the effects of the TRPV1 channel antagonist capsazepine on morphine tolerance and neurotoxicity.

## Conclusion

In conclusion, our findings suggest that TRPV1 receptors play an important role in the modulation of morphine-induced mitochondrial damage and apoptosis in morphine-tolerant rats and in C6 cells exposed to morphine toxicity. The results of this study indicate that the TRPV1 antagonist CPZ may be promising as a therapeutic agent to attenuate the development of morphine tolerance and its associated neurotoxic effects. However, further molecular studies are needed to fully elucidate the role of TRPV1 channels in the antinociceptive tolerance mechanisms of morphine. In addition, further clinical and molecular studies are needed for the use of capsazepine together with morphine to reduce morphine tolerance in patients with severe pain. In the clinic, if the effectiveness of capsazepine is proven, its use together with morphine in patients with severe pain will increase the analgesic effectiveness of morphine, and further dose increases will not be required for morphine. In addition, the incidence of side effects caused by high doses of morphine will decrease in patients.

## Supplementary Information

Below is the link to the electronic supplementary material.

ESM 1 JPEG (136 KB)

## Data Availability

All source data for this work (or generated in this study) are available upon reasonable request.

## Abbreviations

AIF: Apoptosis-inducing factor
ANOVA: One-way analysis of variance
ATCC: American Type Culture Collection
Bcl-2: B-cell lymphoma 2
DMEM: Dulbecco’s Modified Eagle Medium
DMSO: Dimethyl sulfoxide
DRGs: Dorsal root ganglia
ELISA: Enzyme-linked immunosorbent assay
IHC: Immunohistochemical
MOR: Mu-opioid receptor
MORP: Morphine
MPE: Maximum possible effect
MT: Morphine tolerant
NLRP3: NLR family pyrin domain containing 3
OFT: Open field test
PBS: Phosphate-buffered saline
ROS: Reactive oxygen species
TRPM2: Transient receptor potential cation channel, subfamily M, member 2
TRPV1: Transient receptor potential vanilloid type 1
XTT: 2,3-Bis-(2-metoksi-4-nitro-5-sülfofenil)-2H-tetrazolyum-5-karboksianilid
