# Supplementary figures and images for: TRPV1 channel antagonist capsazepine alleviates morphine tolerance and morphine-induced neurotoxicity by preventing mitochondrial damage and apoptosis: an in vivo and in vitro study
Source: Naunyn Schmiedebergs Arch Pharmacol. 2025 Jun 23;398(12):17773–91. doi: 10.1007/s00210-025-04384-5 (PMC12678483; doi:10.1007/s00210-025-04384-5)

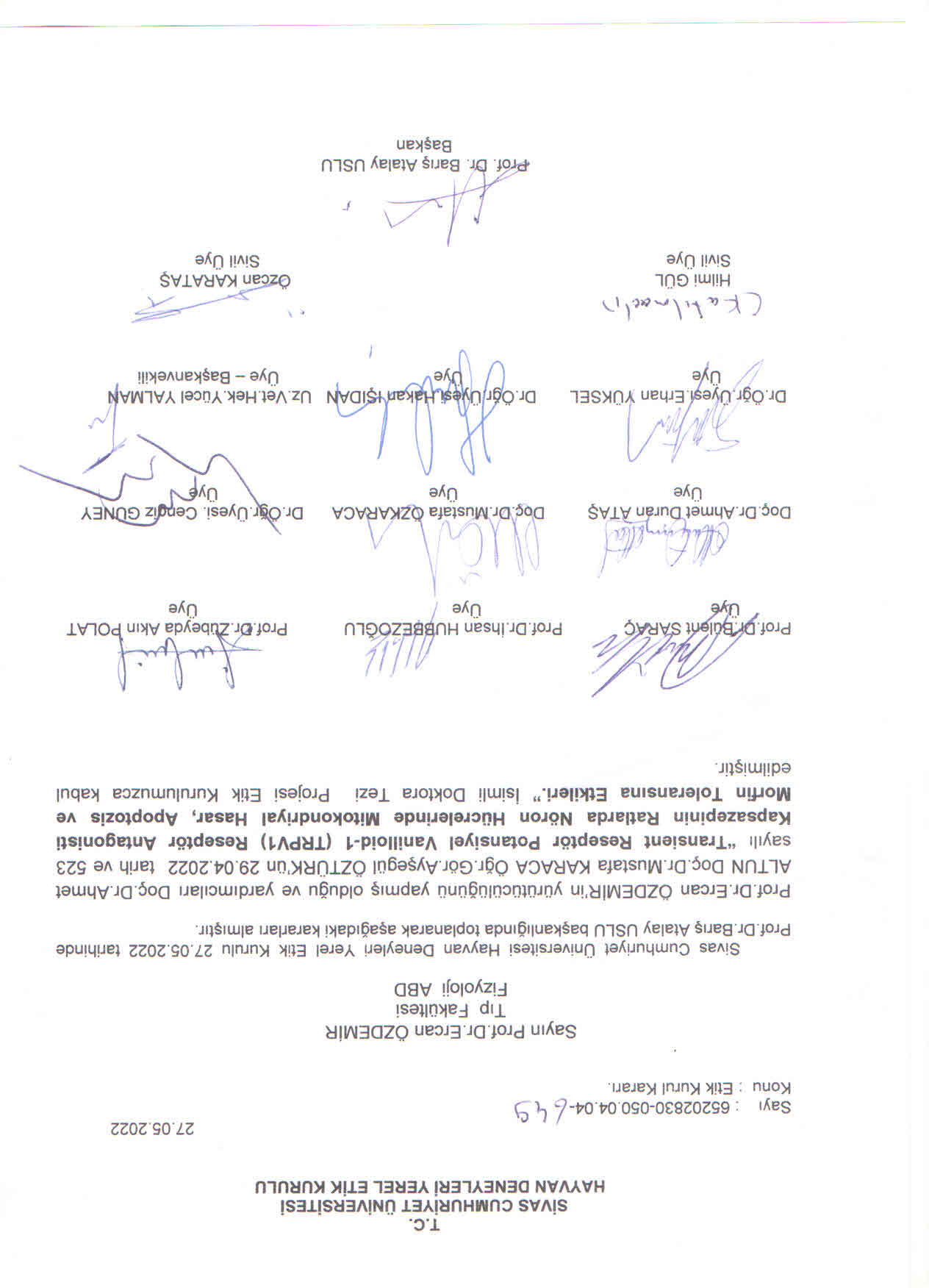

Supplement: Supplementary file 1 — JPEG (136 KB) [file 210_2025_4384_MOESM1_ESM.jpg]
